# ﻿Integrated evidence reveals a new subspecies of *Amolops* Cope, 1865 (Anura, Ranidae) from northeastern Yunnan, China

**DOI:** 10.3897/zookeys.1257.165617

**Published:** 2025-10-24

**Authors:** Pengying Li, Jiangyu Li, Wei Zhang, Xiangbo Yi, Zhiyong Yuan, Junkai Huang, Xiaolong Liu

**Affiliations:** 1 Management and Conservation Bureau of Yunnan Wumeng Mountain National Nature Reserve, Zhaotong 657000, China; 2 Integrative Science Center of Germplasm Creation in Western China (Chongqing) Science City, Biological Science Research Center, Southwest University, Chongqing 400715, China; 3 Key Laboratory of Freshwater Fish Reproduction and Development (Ministry of Education), School of Life Sciences, Southwest University, Chongqing 400715, China; 4 Fuzhou Shuiyuzan Agriculture and Forestry Technology Company, Fuzhou 350100, China

**Keywords:** *Amolops dafangensis wumengmontis* ssp. nov., cascade frogs, species group, subspecies, taxonomy

## Abstract

A new subspecies of *Amolops dafangensis* Li, Liu, Ke, Cheng, & Wang, 2024, designated as *Amolops dafangensis wumengmontis***ssp. nov.**, is described from Mt. Wumeng, Zhaotong City, northeastern Yunnan, China, based on phylogenetic analysis of the 16S rRNA and *CO1* genes, as well as morphological examinations. Phylogenetic analysis indicated that specimens from the Wumeng Mountains clustered within *Amolops dafangensis*, forming two main clades within this species. *Amolops dafangensis wumengmontis***ssp. nov.** demonstrates a close phylogenetic relationship with *A. d. dafangensis* and is differentiated from the nominal subspecies by specific characteristics: 1) tympanum indistinct; 2) tibiotarsal articulation just reaching anterior corner of the eye; 3) flat and indistinct inner and outer metacarpal tubercles; and 4) webbing formula: I1–1II1–1½III1–2IV2–1V.

## ﻿Introduction

The genus *Amolops* Cope, 1865, also known as cascade frogs, comprises 89 recognized species and is widely distributed across southern Asia, from the southern and eastern Himalayas to southeastern mainland China, extending southward to Peninsular Malaysia. China harbors the highest species diversity within this genus, with 60 documented species (Wu et al. 2020; Zeng et al. 2020; AmphibiaChina 2025; Frost 2025). Recent taxonomic revisions, integrating morphological and molecular phylogenetic analyses, have subdivided *Amolops* into eight distinct species groups: the *A. mantzorum* group, *A. monticola* group, *A. hainanensis* group, *A. ricketti* group, *A. marmoratus* group, *A. larutensis* group, *A. daiyunensis* group, and the *A. viridimaculatus* group, based on the amalgamation of morphological and molecular phylogenetic evidence (Cai et al. 2007; Lyu et al. 2019a, 2019b; Wu et al. 2020; Zeng et al. 2020; Jiang et al. 2021; Patel et al. 2021; Saikia et al. 2022a, 2022b).

*Amolops mantzorum* group, established by Fei et al. (1999) based on morphological characteristics, is distinguished from other species groups by a set of unique morphological characteristics, including medium body size, finger I with an expanded tip lacking a circummarginal groove, absence of vocal sac, absence of dorsolateral fold (or presence of glandular fold in some individuals), indistinct but visible tympanum, presence of a nuptial pad on finger I in males, and overlapping heels when hindlimbs are flexed at a right angle (Lyu et al. 2019b; Wu et al. 2020; Tang et al. 2023; Qian et al. 2023; Li et al. 2024). During herpetological surveys in Wumeng Mountain National Nature Reserve, Zhaotong City, northeastern Yunnan, China, a series of specimens belonging to the *A. mantzorum* group were collected, exhibiting similarities in appearance to *A. dafangensis* Li, Liu, Ke, Cheng & Wang, 2024 specimens from Guizhou Province. Subsequent molecular phylogenetic and morphological analyses revealed their close phylogenetic relationship to *A. dafangensis*, albeit with discernible genetic distinctions. Through integrated morphological comparisons, we propose recognizing these specimens as a new subspecies of *A. dafangensis*, designated as *A. d. wumengmontis* ssp. nov.

## ﻿Materials and methods

Fieldwork was conducted in Wumeng Mountain National Nature Reserve, Zhaotong City, northeastern Yunnan, China (Fig. 3). Six specimens were collected in August 2022 and November 2023. Four specimens were euthanized using a low concentration of clove oil solution, and two specimens were released after toe V was clipped to minimize depletion of the wild-sampled population while ensuring individual survival. Liver and muscle tissues were extracted and preserved in 95% ethanol, and the specimens were subsequently fixed in 75% ethanol. Voucher specimens (SWU 0009561, SWU 0009562, SWU 007379, SWU 007381) were deposited at Southwest University (SWU). All sample collections were performed according to the ethical guidelines approved by the Institutional Animal Care and Use Committee (IACUC) of Southwest University (Approval No. LAC2025-2-0113).

### ﻿Morphology and morphometrics

All the measurements were taken using digital slide calipers (accuracy: ± 0.1 mm). Morphological terminology follows Fei et al. (2009) and Lyu et al. (2019b), with detailed definitions provided in Table 1. The webbing formula adheres to the standards of Savage (1975). Complete morphological measurements for all specimens are listed in Table 2. Sex was determined by the presence/absence of nuptial pads on the finger.

**Table 1.** Morphological characters used for adult individuals.

Abbreviation	Morphology
SVL	Snout-vent length
HDL	Head length
HDW	Head width
SL	Snout length
IND	Internarial distance
IOD	Interorbital distance
UEW	Width of upper eyelid
NED	Nasal to eye distance
NSD	Nasal to snout distance
ED	Diameter of eye
TD	Diameter of tympanum
HL	Hand length
FAHL	Length of forearm and hand
LAL	Length of lower arm
LAW	Width of lower arm
HLL	Hindlimb length or leg length
FML	Femur length
TFL	Length of foot and tarsus
FL	Foot length
TBL	Tibia length
TBW	Tibia width

**Table 2.** Measurements (mm) of adult specimens in the type series of *A. d. wumengmontis* ssp. nov.

No.	SWU 0009561	SWU 0009562	SWU 007379	SWU 007381
Sex	Male	Male	Male (holotype)	Female
SVL	43.0	43.3	45.5	62.1
HDL	14.7	14.8	15.7	22.3
HDW	12.4	13.7	12.9	19.0
SL	5.9	5.7	6.7	8.4
IND	5.2	5.0	5.0	6.9
IOD	3.6	4.1	4.4	5.9
UEW	3.2	2.8	4.0	4.5
NED	2.8	1.9	2.6	4.0
NSD	3.0	3.2	3.4	5.0
ED	5.4	5.5	5.6	7.1
TD	1.6	1.9	1.5	2.3
HL	13.1	13.5	13.3	18.2
FAHL	20.8	21.3	22.3	30.1
LAL	7.7	7.8	9.0	11.9
LAW	4.7	4.7	4.8	5.3
HLL	77.0	78.0	80.7	110.3
FML	24.1	26.5	25.5	33.8
TFL	34.8	35.6	36.2	47.5
FL	22.5	22.9	22.3	32.9
TBL	25.9	26.7	26.2	33.1
TBW	5.0	4.9	5.8	7.3

### ﻿DNA sequencing and molecular analyses

To construct a phylogeny for the *A. mantzorum* group, we extracted total DNA from liver or muscle tissue using the Animal Tissue DNA Isolation Kit provided by Thermo Fisher Scientific. In this study, we sequenced two mitochondrial genes: 16S rRNA and *CO1*. The primers used for polymerase chain reaction (PCR) amplification are detailed in Suppl. material 1. The PCR amplification process was carried out in a 20 μL reaction volume, following the reaction cycling settings below: an initial denaturation step at 95 °C for 4 min; followed by 36 cycles of denaturation at 95 °C for 30 s, annealing at 54 °C (for 16S rRNA)/ 49 °C (for *CO1*) for 40 s, and extension at 72 °C for 70 s, and all sequences have been uploaded to GenBank (Table 3). Based on Tang et al. (2023), we selected species from other species groups of *Amolops*, *Amolops chaochin* Jiang, Ren, Lyu & Li, 2021 and *A. ricketti* (Boulenger, 1899) as outgroups to reconstruct the phylogenetic relationships of the *A. mantzorum* group; all GenBank accession numbers are listed in Table 3.

**Table 3.** *Amolops* species used in phylogenetic analyses of this study; “/” means unknown.

ID	Species	Voucher ID	Locality	GenBank accession number	
				16S rRNA	CO1
1	*A. d. wumengmontis***ssp. nov**.	SWU 0009561	Mt. Wumeng, Zhaotong, Yunnan, China	PX411479	PX410826
2	*A. d. wumengmontis* **ssp. nov.**	SWU 0009562	Mt. Wumeng, Zhaotong, Yunnan, China	PX411478	PX410827
3	*A. d. wumengmontis* **ssp. nov.**	SWU 007379	Yiliang, Zhaotong, Yunnan, China	PX411483	PX410822
4	*A. d. wumengmontis* **ssp. nov.**	SWU 007381	Yiliang, Zhaotong, Yunnan, China	PX411482	PX410823
5	*A. d. wumengmontis* **ssp. nov.**	Tissue ID: Yuan 30620	Yiliang, Zhaotong, Yunnan, China	PX411481	PX410824
6	*A. d. wumengmontis* **ssp. nov.**	Tissue ID: Yuan 30621	Yiliang, Zhaotong, Yunnan, China	PX411480	PX410825
7	*A. sangzhiensis*	CSUFT 901	Mt. Doupeng, Sangzhi, Hunan, China	OQ079538	OQ078903
8	*A. sangzhiensis*	CSUFT 905	Mt. Doupeng, Sangzhi, Hunan, China	OQ079539	OQ078904
9	*A. sangzhiensis*	CSUFT 907	Mt. Doupeng, Sangzhi, Hunan, China	OQ079540	OQ078905
10	*A. sangzhiensis*	CSUFT 912	Mt. Doupeng, Sangzhi, Hunan, China	OQ079541	OQ078906
11	*A. sangzhiensis*	CSUFT 916	Mt. Doupeng, Sangzhi, Hunan, China	OQ079542	OQ078907
12	*A. sangzhiensis*	CSUFT 927	Mt. Doupeng, Sangzhi, Hunan, China	OQ079543	OQ078908
13	*A. granulosus*	SYS a005399	Mt. Guangwu, Sichuan, China	MK573811	MK568326
14	*A. granulosus*	SYS a005400	Mt. Guangwu, Sichuan, China	MK573812	MK568327
15	*A. granulosus*	SYS a005318	Mt. Wawu, Sichuan, China	MK573802	MK568317
16	*A. granulosus*	SYS a005319	Mt. Wawu, Sichuan, China	MK573803	MK568318
17	*A. granulosus*	SCUM 045823HX	Dayi, Sichuan, China	MN953680	JN700804
18	*A. jinjiangensis*	SYS a004571	Mt. Gaoligong, Yunnan, China	MK573801	MK568316
19	*A. jinjiangensis*	SCUM 050434CHX	Deqing, Yunnan, China	MN953700	MN961402
20	*A. jinjiangensis*	SCUM 050435CHX	Deqing, Yunnan, China	EF453741	MN961403
21	*A. lifanensis*	SYS a005374	Lixian, Sichuan, China	MK573809	MK568324
22	*A. lifanensis*	SYS a005375	Lixian, Sichuan, China	MK573810	MK568325
23	*A. lifanensis*	SYS a005376	Lixian, Sichuan, China	MK604868	MK605626
24	*A. lifanensis*	SYS a005377	Lixian, Sichuan, China	MK604869	MK605627
25	*A. lifanensis*	SYS a005378	Lixian, Sichuan, China	MK604870	MK605628
26	*A. loloensis*	SYS a005346	Zhaojue, Sichuan, China	MK604854	MK605612
27	*A. loloensis*	SYS a005347	Zhaojue, Sichuan, China	MK604855	MK605613
28	*A. loloensis*	SYS a005348	Zhaojue, Sichuan, China	MK604856	MK605614
29	*A. loloensis*	SYS a005349	Zhaojue, Sichuan, China	MK604857	MK605615
30	*A. loloensis*	SCUM 045806HX	Xichang, Sichuan, China	MN953704	MN961407
31	*A. loloensis*	SCUM 045807HX	Xichang, Sichuan, China	EF453743	MN961456
32	*A. mantzorum*	SYS a005365	Fengtongzhai, Sichuan, China	MK573808	MK568323
33	*A. mantzorum*	SYS a005366	Fengtongzhai, Sichuan, China	MK604862	MK605620
34	*A. mantzorum*	SYS a005367	Fengtongzhai, Sichuan, China	MK604863	MK605621
35	*A. mantzorum*	SYS a005368	Fengtongzhai, Sichuan, China	MK604864	MK605622
36	*A. mantzorum*	SYS a005370	Fengtongzhai, Sichuan, China	MK604865	MK605623
37	*A. mantzorum*	SYS a005371	Fengtongzhai, Sichuan, China	MK604866	MK605624
38	*A. mantzorum*	SYS a005372	Fengtongzhai, Sichuan, China	MK604867	MK605625
39	*A. mantzorum*	SYS a005356	Kangding, Sichuan, China	MK604858	MK605616
40	*A. mantzorum*	SYS a005357	Kangding, Sichuan, China	MK604859	MK605617
41	*A. mantzorum*	SYS a005358	Kangding, Sichuan, China	MK604860	MK605618
42	*A. mantzorum*	SYS a005336	Mt. Wawu, Sichuan, China	MK573804	MK568319
43	*A. mantzorum*	SYS a005337	Mt. Wawu, Sichuan, China	MK604853	MK605611
44	*A. mantzorum*	SCUM 045817HX	Wolong, Sichuan, China	MN953706	MN961408
45	*A. mantzorum*	SCUM 045825HX	Dayi, Sichuan, China	MN953707	MN961409
46	*A. mantzorum xinduqiao*	KIZ 041127	Kangding, Sichuan, China	MN953764	MN961465
47	*A. mantzorum xinduqiao*	KIZ 041129	Kangding, Sichuan, China	MN953765	MN961466
48	*A. minutus*	KIZ 2023068	Yuanyang, Yunnan, China	PQ346031	/
49	*A. minutus*	KIZ 2023069	Yuanyang, Yunnan, China	PQ346032	/
50	*A. minutus*	KIZ 2023070	Yuanyang, Yunnan, China	PQ346033	/
51	*A. shuichengicus*	SYS a004956	Shuicheng, Guizhou, China	MK604845	MK605603
52	*A. shuichengicus*	SYS a004957	Shuicheng, Guizhou, China	MK604846	MK605604
53	*A. shuichengicus*	SYS a004958	Shuicheng, Guizhou, China	MK604847	MK605605
54	*A. shuichengicus*	SYS a004971	Shuicheng, Guizhou, China	MK604848	MK605606
55	*A. tuberodepressus*	SCUM 050430CHX	Jingdong, Yunnan, China	MN953730	MN961433
56	*A. tuberodepressus*	SCUM 050433CHX	Jingdong, Yunnan, China	MN953729	MN961432
57	*A. tuberodepressus*	SYS a003900	Mt. Ailao, Yunnan, China	MK573797	MK568314
58	*A. tuberodepressus*	SYS a003901	Mt. Ailao, Yunnan, China	MK573798	MK568315
59	*A. tuberodepressus*	SYS a003902	Mt. Ailao, Yunnan, China	MK604844	MK605602
60	*A. tuberodepressus*	SYS a003931	Mt. Wuliang, Yunnan, China	MK573799	MG991933
61	*A. ailao*	GXNU YU000001	Mt. Ailao, Yunnan, China	MN650751	MN650737
62	*A. ailao*	GXNU YU000002	Mt. Ailao, Yunnan, China	MN650752	MN650738
63	*A. ailao*	GXNU YU000003	Mt. Ailao, Yunnan, China	MN650753	MN650739
64	*A. ailao*	GXNU YU000004	Mt. Ailao, Yunnan, China	MN650754	MN650740
65	*A. d. dafangensis*	MT DF20230601002	Dafang, Guizhou, China	OR936315	OR924345
66	*A. d. dafangensis*	MT DF20230601003	Dafang, Guizhou, China	OR936316	OR924346
67	*A. d. dafangensis*	MT DF20230601004	Dafang, Guizhou, China	OR936317	OR924347
68	*A. chaochin*	XM5526	Wenxian, Gansu, China	KX645666	KX645666
69	*A. ricketti*	SYS a004141	Mt. Wuyi, Fujian, China	MK263259	MG991927

Sequences were aligned using the MUSCLE option in MEGA v. 7 (Kumar et al. 2016). Before phylogenetic reconstruction, the best substitution model was selected using the Akaike Information Criterion (AIC) in jMODELTEST v. 2.1.10 (Darriba et al. 2012). Bayesian inference (BI) was performed using MrBayes v. 3.2.6 (Ronquist et al. 2012), with two runs performed simultaneously, each consisting of four Markov chains starting from a random tree. The chain was run for 6,000,000 generations, with sampling every 1000 generations. When the average standard deviation of the split frequency was less than 0.01, the first 25% of the sampled trees were discarded as burn-in, and the remaining trees were used to create a consensus tree and estimate the Bayesian posterior probability. Maximum likelihood (ML) analyses were performed using RAxML v. 7.0.3 (Stamatakis 2014) under the GTRGAMMA model with 1000 bootstrap replicates.

We used Assemble Species by Automatic Partitioning (ASAP) for species delimitation. For this method, we used the simple distance (*p*-distance) model, and the partition with the lowest ASAP*p*-score was deemed the best (Puillandre et al. 2021). Additionally, due to the excessively small genetic distances in 16S rRNA among species within the *A. mantzorum* group, the ASAP could not accurately delimit species; we only selected the *CO1* gene for species delimitation.

## ﻿Results

### ﻿Phylogenetic analyses

**Figure 1.**
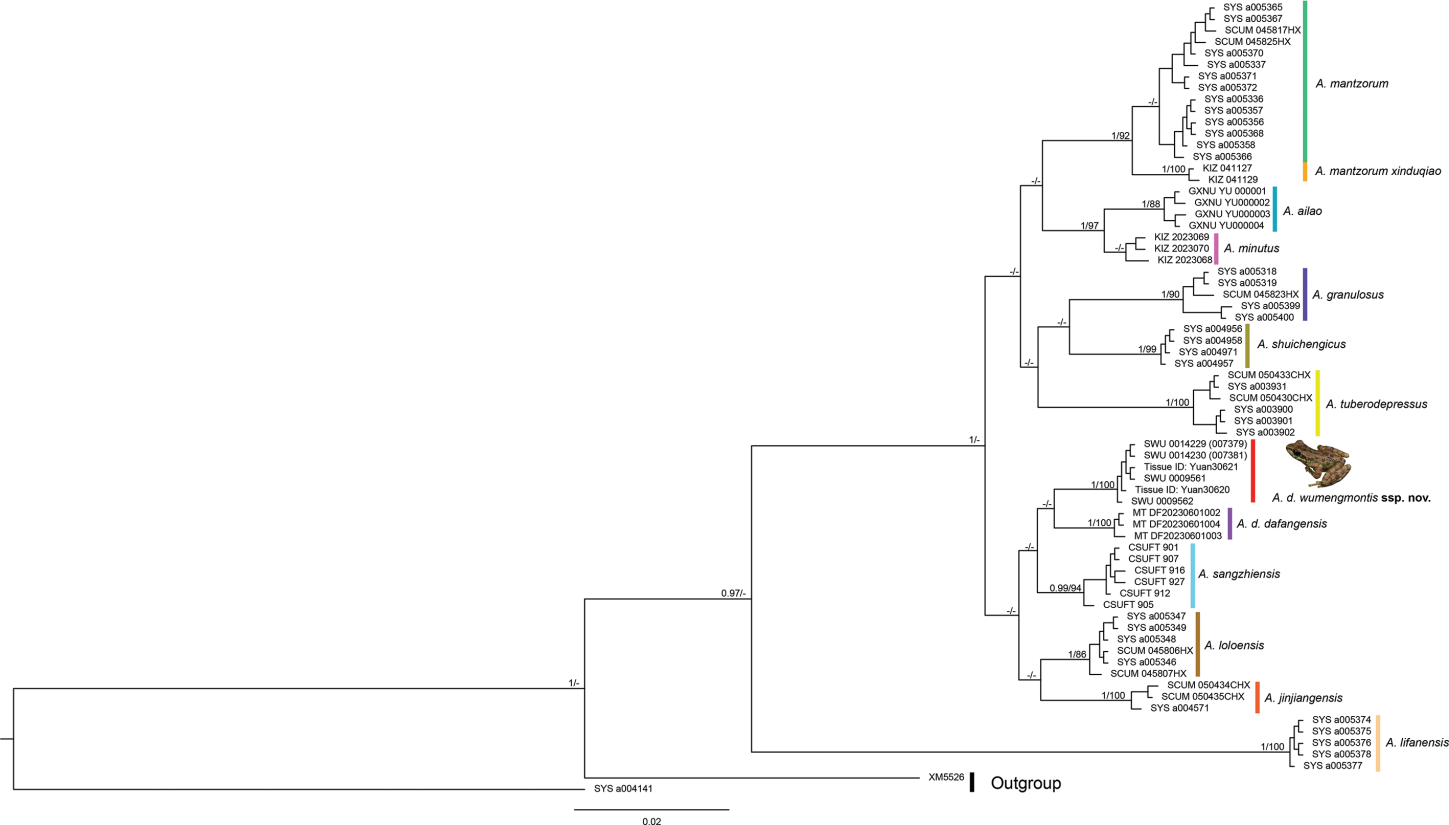
Bayesian phylogram of *Amolops mantzorum* group inferred from the combination of 16S rRNA and *CO1* genes. The numbers above and below the branches are Bayesian posterior probabilities (BPP) and maximum likelihood bootstrap values (BS); “-” denotes a BPP < 0.95 and BS < 70.

**Figure 2.**
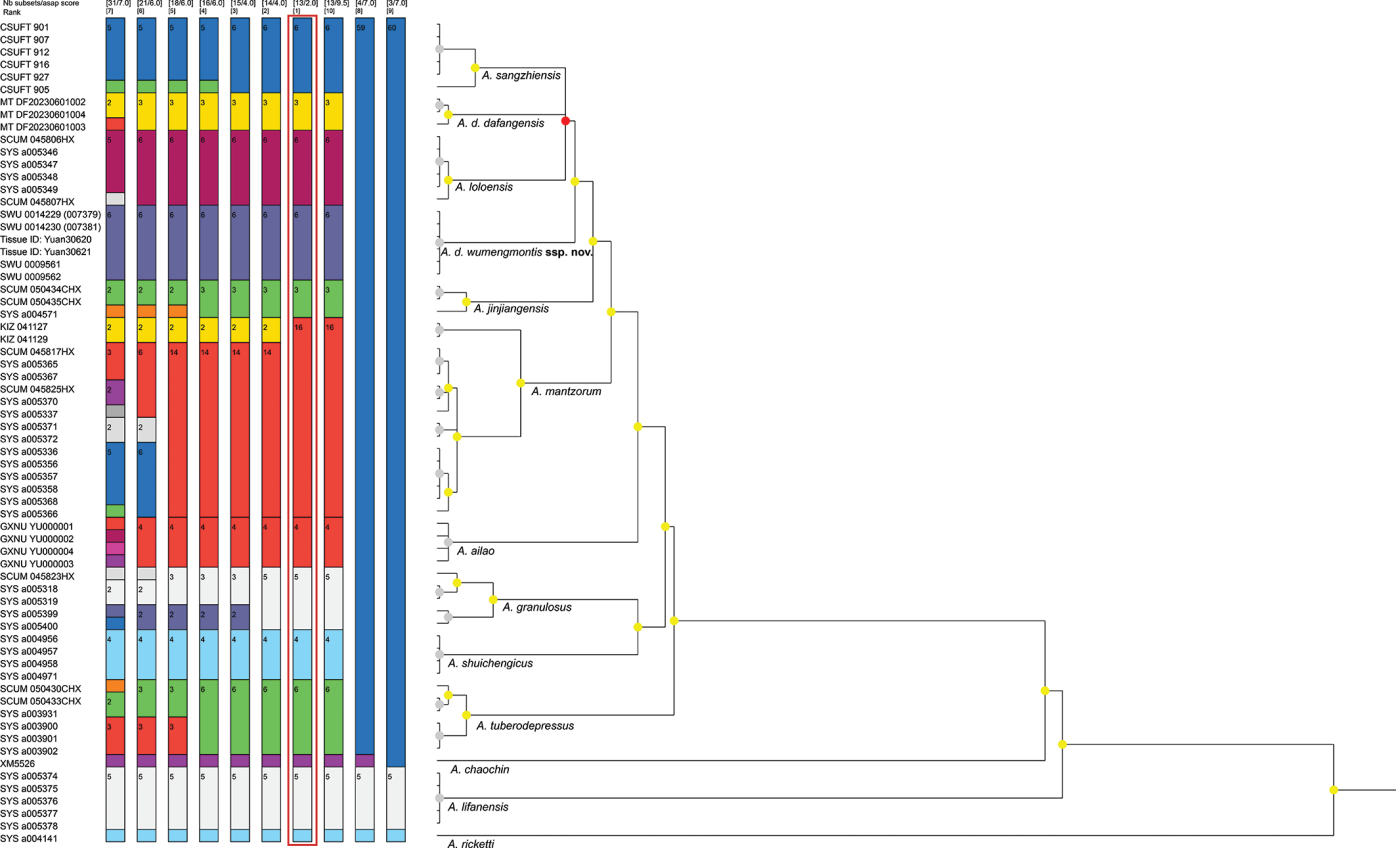
ASAP species delimitation based on *CO1* sequences. The best partition with the lowest score is highlighted with a red frame.

The length of the sequence alignment was 1094 base pairs. Phylogenetic trees generated using BI and ML methods exhibit overall congruence (Fig. 1). In contrast, the species delimitation results based on ASAP analysis of *CO1* sequences differed from the phylogenetic tree topology (Fig. 2). Mean p-distances for the 16S rRNA and *CO1* genes among the *Amolops* species used in this study are provided in Suppl. material 2. Phylogenetic analysis indicated that specimens from the Wumeng Mountains clustered within *Amolops dafangensis*, forming two main clades within this species. The species delimitation results based on ASAP analysis of *CO1* sequences indicate that the specimens from the Wumeng Mountains form a sister group with *A. sangzhiensis* Qian, Xiang, Jiang, Yang & Gui, 2023 and *A. loloensis* (Liu, 1950), suggesting that they should be recognized as a distinct species. The *p*-distance between specimens from the Wumeng Mountains and *A. dafangensis* is 0.5% on the 16S rRNA gene and 2.8% on the *CO1* gene.

**Figure 3.**
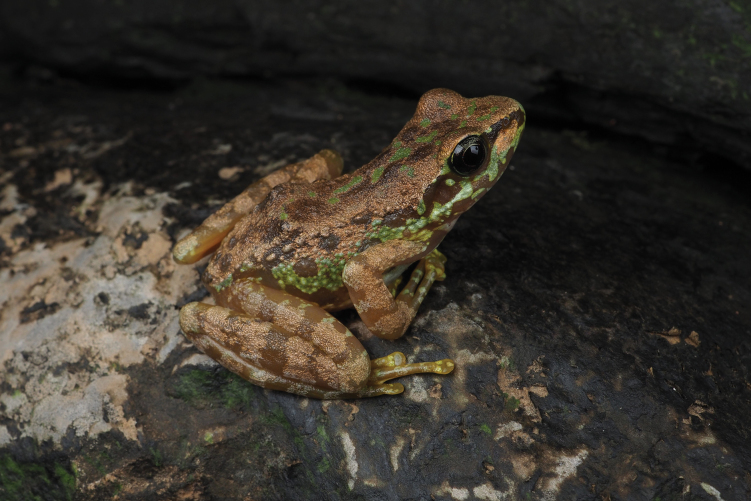
Holotype SWU 007379 of *A. d. wumengmontis* ssp. nov. in situ.

### ﻿Taxonomic account

#### ﻿ Amolops dafangensis wumengmontis

Taxon classificationAnimaliaAnuraRanidae

Huang, Yuan & Liu ssp. nov.

40758055-DE6D-55DB-8C5C-8043222C0DFD

https://zoobank.org/AA78B643-6A58-4A8D-9298-0CC7E169572C

Figs 3, 5; Table 2

##### Holotype.

**Figure 4.**
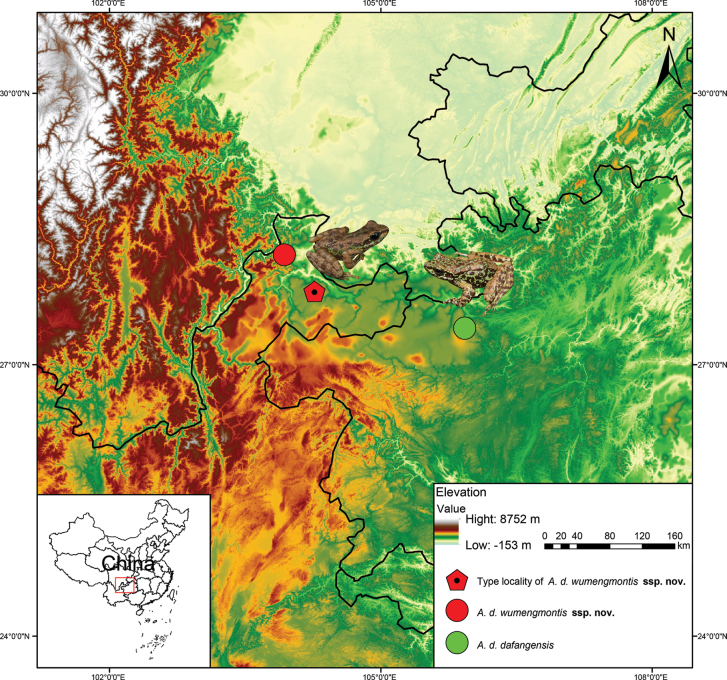
Distribution localities of *A. d. wumengmontis* ssp. nov. in Wumeng Mountains, northeastern Yunnan, China.

• SWU 007379, adult male (Fig. 3), collected in July 2021 by Xiaolong Liu and Rui Chen from Wumeng Mountain National Nature Reserve, Zhaotong City, northeastern Yunnan, China (27.810588°N, 104.266768°E; 1915 m a.s.l.) (Fig. 4).

##### Paratypes.

• Two adult males (SWU 0009561, SWU 0009562) were collected at Wumeng Mountain National Nature Reserve, Zhaotong City, northeastern Yunnan, China (28.213406°N, 103.935201°E; elevation 1797 m a.s.l.) in November 2023 by Xiaolong Liu and Rui Chen. • One adult female (SWU 007381) was collected at the same locality as the holotype in July 2021 by Xiaolong Liu and Rui Chen.

##### Etymology.

The specific epithet “wumengmontis” is derived from the type locality, Wumeng Mountain National Nature Reserve, Zhaotong City, northeastern Yunnan, China. We suggest ‘Wumeng Cascade Frog’ as its English common name, and ‘Dà Fāng Tuān Wā Wū Mēng Yà Zhŏng’ (大方湍蛙乌蒙亚种) as its Chinese common name.

##### Diagnosis.

*Amolops dafangensis wumengmontis* ssp. nov. was classified within the genus *Amolops* and further assigned to the *A. mantzorum* group due to the combination of following features: 1) moderate body size, with a SVL of 43.0–45.5 mm in adult males (*N* = 3) and 62.1 mm in adult female (*N* = 1); 2) presence of vomerine teeth; 3) slightly longer head length compared to head width; 4) indistinct tympanum; 5) lack of webbing or lateral fringes on fingers, as well as absence of lateral fringes on toes; 6) maxillary glands extremely indistinct, appearing as three small clusters; 7) presence of supratympanic folds and dorsolateral folds formed by a series of glands; 8) tibiotarsal articulation reaching the anterior corner of the eye; 9) heels overlapping when hindlimbs flexed at right angles to the body axis; and 10) absence of vocal sacs in males, with nuptial pads featuring velvety white nuptial spines at the base of finger I.

##### Description of holotype.

Adult male, body size moderate (SVL = 45.5 mm) with a head length slightly surpassing head width (HDL/HDW = 1.2). The snout is moderately rounded, the canthus rostralis is well-defined and curved, while the loreal region slopes concavely. The nostrils are circular, positioned between the snout and eyes, and slightly protruding. The eyes are large (ED/SL = 0.8), the internarial distance exceeds the interorbital distance (IND/IOD = 1.1), and the width of the upper eyelid is slightly narrower than the interorbital distance (UEW/IOD = 0.9). A discernible pineal spot is present, the pupil is round and horizontally oriented, and the mouth corner is smooth. The tympanum is indistinct, with maxillary glands appearing extremely indistinct, resembling three small clusters. There is an indistinct supratympanic fold, vomerine teeth were observed, choanae are round, the tongue is anteriorly attached and cordiform, with a notched posterior margin, and a vocal sac is notably absent.

The forelimbs are robust, with the relative length order of fingers being I < II < IV < III; all fingers are expanded into discs, with circummarginal grooves present on all except finger I. There is no webbing or lateral fringes on the fingers. Nuptial pads featuring velvety white nuptial spines are located at the base of finger I. Webbing between the fingers is absent. The subarticular tubercles are distinct and rounded, supernumerary tubercles are present, and both the inner and outer metacarpal tubercles are flat and indistinct.

The hindlimbs are elongated, nearly twice the snout-vent length (HLL/SVL = 1.8), with the tibiotarsal articulation reaching the anterior corner of the eye. When the hindlimbs are flexed at right angles to the body axis, the heels overlap. The tibia length exceeds the combined lengths of the forearm and hand (TBL/FAHL = 1.2). The relative length order of the toes is I < II < III < V < IV, with all toe tips expanded into discs bearing circummarginal grooves. Well-developed webbing is present between the toes, covering two-thirds of the interdigital spaces, following the webbing formula: I1–1II1–1½III1–2IV2–1V. There are no lateral fringes on the toes. The subarticular tubercles are distinct, the inner metatarsal tubercle is rounded, and the outer metatarsal tubercle is absent. Supernumerary tubercles were not observed.

Skin on the dorsum and dorsal surfaces of limbs is smooth. Weak dorsolateral folds, formed by a series of glands and appearing as incomplete lines (glandular dorsolateral folds), extend from above the shoulder to the vent. Weak dorsolateral glandular lines are present. Prominent folds are noticeable around the anus.

***Coloration of the holotype in life*.** In the holotype specimen observed in life (see Fig. 3), the iris appears black with a brown wash. The dorsal surface exhibits a golden-brown color with rounded black-brown and green spots. A pale green, irregular stripe runs from the snout to the vent along the flank of the body. The temporal region is characterized by black-brown hues interspersed with green and golden-brown spots. The chest and abdomen are predominantly white with pale brown markings. The ventral surface of the forelimbs displays a light brown coloration with green spots on the outer side and white on the inner side, while all the discs are yellow-green. The webbing between the toes and the ventral surface of the hindlimbs presents a yellow coloration.

**Figure 5.**
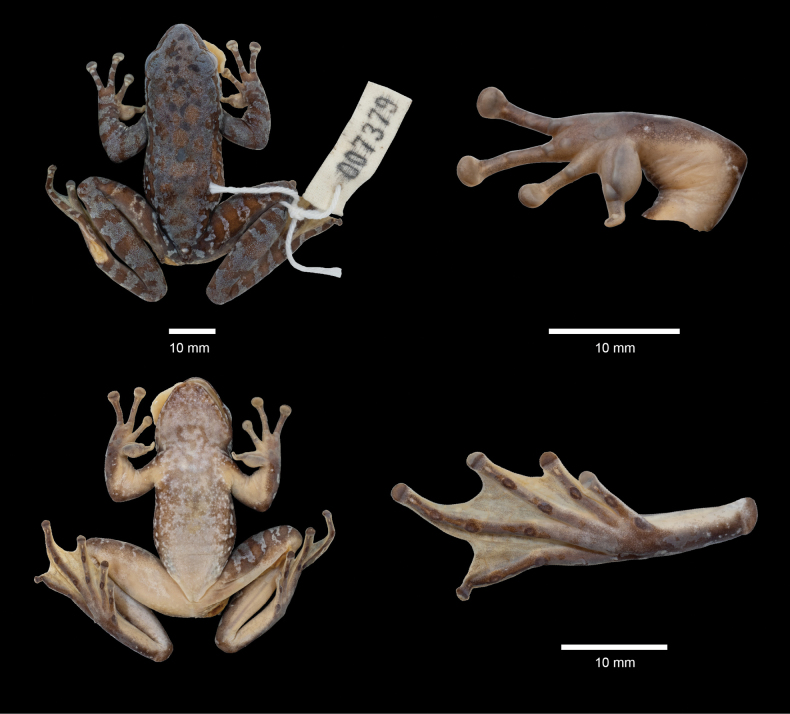
Holotype SWU 007379 of *A. d. wumengmontis* ssp. nov. in preservative.

***Coloration of the holotype in preservative*.** After preservation in alcohol, the coloration of the specimen has faded, although the general pattern remains unchanged. Dorsal color has transitioned to a grey-blue and dark brown hue, while the ventral surface has faded to a milky white shade. The webbing between the toes and the ventral surface of the hindlimbs has also faded to a brown color (Fig. 5).

##### Sexual dimorphism.

Males exhibit a smaller size compared to females; they are devoid of vocal sacs, and nuptial pads featuring velvety white nuptial spines are present at the base of finger I.

##### Variation.

**Figure 6.**
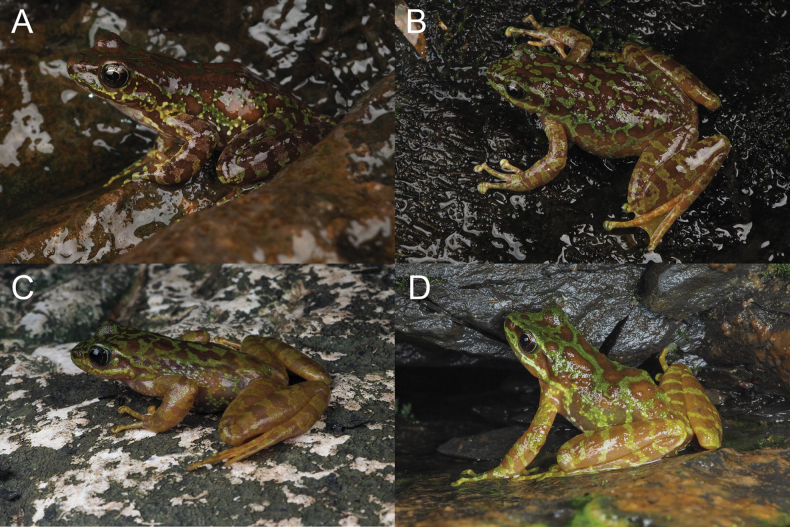
Different coloration of *A. d. wumengmontis* ssp. nov. A. Tissue ID: Yuan30621; B. SWU 007381; C. SWU 009561; D. SWU 0009562.

All specimens displayed a high degree of morphological similarity; there was variability in the coloration among live individuals (Fig. 6).

##### Distribution and ecology.

**Figure 7.**
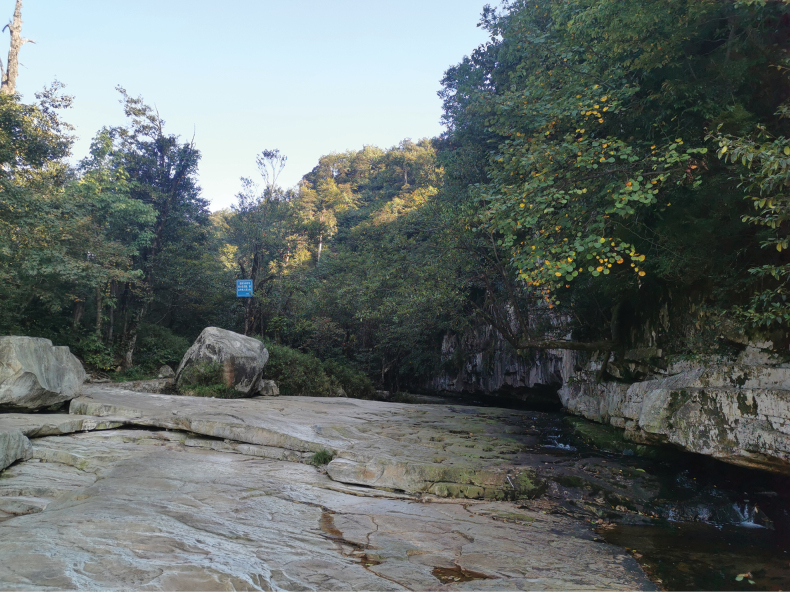
Habitat of *A. d. wumengmontis* ssp. nov. at the type locality, Wumeng Mountain National Nature Reserve, Zhaotong City, northeastern Yunnan, China.

This species is currently known to be distributed only in stream areas above 1500 m elevation in the Wumeng Mountains (Yongshan County, Yiliang County) (Fig. 7). This species is sympatric with *Leptobrachium boringii* (Liu, 1945) and *Odorrana margaretae* (Liu, 1950). The male individuals collected in July had nuptial pads, but no tadpoles or amplexus behaviors were observed, indicating an early stage of the breeding season.

##### Comparisons.

Instead of comparing the new subspecies of *A. dafangensis* to all known *Amolops*, our focus is on the morphological distinctions in comparison to phylogenetically closely related taxa, specifically *A. dafangensis dafangensis* (Li et al. 2024) in the *A. mantzorum* group (Table 4). The new subspecies resembles *A. dafangensis dafangensis* in the following morphological characteristics: 1) moderate body size, with a SVL of 43.0–45.5 mm in adult males (*N* = 3); 2) slightly longer head length compared to head width; 3) absence of vocal sacs in males, with nuptial pads featuring velvety white nuptial spines at the base of finger I; 4) presence of vomerine teeth; and 5) presence of supratympanic folds and dorsolateral folds formed by a series of glands. However, the new subspecies can be distinguished from *A. dafangensis dafangensis* based on the following morphological characteristics: 1) indistinct tympanum (vs. distinct tympanum); 2) tibiotarsal articulation extending just to the anterior corner of the eye (vs. extending far beyond the tip of the snout); 3) flat and indistinct inner and outer metacarpal tubercles (vs. oval inner metacarpal tubercle and small, round outer metacarpal tubercles); and 4) webbing formula: I1–1II1–1½III1–2IV2–1V (vs. webbing formula: I1–1II1–1½III1–2½IV2½–1V).

**Table 4.** Comparison of *A. d. wumengmontis* ssp. nov. with species in the *A. mantzorum* group; “/” means unknown.

Species	A. d. wumengmontis ssp. nov.	A. d. dafangensis
Adult male	43.0–45.5 mm	43.2–46.8 mm
Adult female	62.1 mm	/
HDL/HDW	Head length slightly longer than head width	Head length slightly longer than head width
Tympanum	Indistinct	Distinct
Canthus rostralis	Distinct	Distinct
Vomerine teeth	Present	Present
Vocal sac	Absent	Absent
Nuptial pad	Velvety nuptial pads present on finger I	Velvety nuptial pads present on finger I
Dorsolateral fold	Glandular dorsolateral fold	Glandular dorsolateral fold
Lateral fringes	Absent	Absent
Finger formula	I < II < IV < III	I < II < IV < III
Toe formula	I < II < III < V < IV	I < II < III < V < IV
Tibiotarsal articulation	Reaching anterior corner of the eye	Far beyond the tip of the snout

## ﻿Discussion

Phylogenetic analyses revealed that *A. dafangensis* from the Wumeng Mountain National Nature Reserve formed a distinct clade separate from the population at the type locality, with a certain degree of genetic divergence (0.5% on the 16S rRNA gene; 2.8% on the *CO1* gene). Relatively stable morphological differences were also observed between these two lineages. Species delimitation based on the ASAP method supported the recognition of the Wumeng population as a distinct species. These findings indicate that they cannot be classified as *A. dafangensis*. It is hypothesized that the Wumeng Mountains may have served as a geographical barrier, restricting gene flow and thereby facilitating the formation of two independent populations. The relatively small genetic distance between the two lineages and the limited number of reliable morphological distinctions led us to avoid oversplitting and instead designate the lineage as a new subspecies, *A. d. wumengmontis* ssp. nov. By designating the samples from the *A. mantzorum* group in the Wumeng Mountains region as *A. d. wumengmontis* ssp. nov., we not only avoid destabilizing the taxonomic system due to excessive species splitting, but also effectively document populations with unique genetic and ecological characteristics, thereby preventing the loss of genetic diversity.

*Amolops mantzorum xinduqiao* Fei, Ye, Wang & Jiang, 2017 (previously *A. xinduqiao*) constitutes a relatively monophyletic clade within the *A. mantzorum* lineage, resulting in *A. mantzorum* (David, 1871) comprising two distinct clades. These clades demonstrate some degree of geographical isolation, with the *A. mantzorum* clade inhabiting the Dadu River basin at mid to high altitudes (1200–2400 m) and the *A. mantzorum xinduqiao* clade occupying the Yalong River basin at high altitudes (above 3000 m). Despite showing low genetic differentiation (0.4% on the 16S rRNA gene, 1.8% on the *CO1* gene), *A. mantzorum xinduqiao* can only be differentiated from *A. mantzorum* based on body color patterns and body size, lacking consistent diagnostic traits (Fei et al. 2017). Our findings support prior studies classifying *A. xinduqiao* as a subspecies of *A. mantzorum* (Dufresnes and Litvinchuk 2022; Tang et al. 2023).

## Supplementary Material

XML Treatment for Amolops dafangensis wumengmontis

## ﻿Additional information

### Use of AI

No use of AI was reported.

### Author ORCIDs

Pengying Li https://orcid.org/0009-0008-5800-1475

Jiangyu Li https://orcid.org/0009-0001-1637-6083

Wei Zhang https://orcid.org/0009-0009-8820-2339

Xiangbo Yi https://orcid.org/0009-0005-9219-853X

Zhiyong Yuan https://orcid.org/0000-0001-5991-3021

Junkai Huang https://orcid.org/0000-0001-5657-9056

Xiaolong Liu https://orcid.org/0000-0002-8417-5208

### Data availability

All of the data that support the findings of this study are available in the main text or Supplementary Information.

## Supplementary materials

Supplementary material 1 Primer pairs for PCR amplification used in this study Data type xlsx https://binary.pensoft.net/file/1448224This dataset is made available under the Open Database License (http://opendatacommons.org/licenses/odbl/1.0/). The Open Database License (ODbL) is a license agreement intended to allow users to freely share, modify, and use this Dataset while maintaining this same freedom for others, provided that the original source and author(s) are credited.Pengying Li, Jiangyu Li, Wei Zhang, Xiangbo Yi, Zhiyong Yuan, Junkai Huang, Xiaolong Liu

Supplementary material 2 Mean uncorrected pairwise distances of 16S rRNA and *CO1* (%) between clades of *A. mantzorum* group Data type xlsx https://binary.pensoft.net/file/1448225This dataset is made available under the Open Database License (http://opendatacommons.org/licenses/odbl/1.0/). The Open Database License (ODbL) is a license agreement intended to allow users to freely share, modify, and use this Dataset while maintaining this same freedom for others, provided that the original source and author(s) are credited.Pengying Li, Jiangyu Li, Wei Zhang, Xiangbo Yi, Zhiyong Yuan, Junkai Huang, Xiaolong Li
